# A Study on a Cast Steel Reinforced with WC–Metal Matrix Composite

**DOI:** 10.3390/ma15186199

**Published:** 2022-09-06

**Authors:** Aida B. Moreira, Laura M. M. Ribeiro, Pedro Lacerda, Ana M. P. Pinto, Manuel F. Vieira

**Affiliations:** 1 Department of Metallurgical and Materials Engineering, University of Porto, R. Dr. Roberto Frias, 4200-465 Porto, Portugal; 2 LAETA/INEGI—Institute of Science and Innovation in Mechanical and Industrial Engineering, R. Dr. Roberto Frias, 4200-465 Porto, Portugal; 3 FERESPE, Fundição de Ferro e Aço Lda, R. Basileia, 4760-485 Vila Nova de Famalicão, Portugal; 4 CMEMS—Center for MicroElectroMechanics Systems, Department of Mechanical Engineering, University of Minho, 4800-058 Guimarães, Portugal

**Keywords:** casting, ex situ technique, local reinforcement, low carbon steel, metal matrix composite, tungsten carbide

## Abstract

This study seeks to investigate the local reinforcement of low carbon cast steel specimens with WC–metal matrix composites (WC–MMCs), to obtain a new material effective in competing with hard alloy steels. For this purpose, a powder compact of tungsten carbide (WC) and iron (Fe) was prepared and placed in the mold cavity before casting. The reactions that occurred with the molten steel led to the formation of the WC–MMC and, consequently, to the local reinforcement of the steel. The microstructure of the WC–MMC reinforcement was characterized by scanning electron microscopy (SEM) with energy dispersive spectroscopy (EDS), X-ray diffraction (XRD), and electron backscatter diffraction (EBSD). The results showed a microstructural variation throughout the depth of the reinforcement. In the surface region, most of the original WC particles retain their polygonal morphology, but towards the base metal, the dissolution of the WC particles increased with the formation of (Fe,W)_6_C carbides. Closer to the base metal, dendritic eutectic carbides of (Fe,W)_6_C and fine (Fe,W)_23_C_6_ precipitates in a matrix of martensite were formed. The mechanical properties of the reinforcement were evaluated by hardness and ball-cratering abrasion tests. The results revealed a significant increase in hardness, being three times harder than the base metal, and a decrease of 39% in the wear rate.

## 1. Introduction

Low carbon cast steels contain less than 0.2 wt.% carbon and alloying elements in a concentration that can reach a value of 8.0 wt.% [1]. Typically, non-alloyed low carbon steels are produced with the nominal composition of 0.16% C, 0.50% Mn, 0.05% P, 0.06% S, and 0.35% Si [1,2,3]. Among other applications, such steels are usually used on metallic structures, components for the automotive industry, pumps and valves for the oil and gas industry, and pressure vessels for energy production, mainly due to their manufacturing facility, low cost, mechanical properties, and weldability [4,5,6]. However, low carbon cast steels have low wear resistance and, therefore, under wear conditions, they have to be replaced by alloyed steels, which are costly. Alternatively, a surface heat treatment, a thermal spray coating, or a less traditional approach with a metal matrix composite (MMC) reinforcement can be applied to improve their surface properties [7,8]. The development of MMC reinforcements in components produced by casting is particularly attractive since it can lead to the surface wear resistance increase by one-step processing [7,8]. Such approach is easy to perform and can be applied to components with any geometry or dimensions, in addition, it is highly efficient and requires low-cost investment [2,3,9,10]. Despite the existing literature providing several studies on the reinforcement of Cr-alloyed steel [11,12,13,14,15], Mn-steel [16,17,18], and medium carbon steel [19], only four studies have described the process of locally reinforcing low carbon steel castings and all with TiC–MMC [20,21,22,23]. Sui et al. [14] studied Cr-alloyed steel reinforced with a surface composite, a mixture of WC particles and Ni60WC25 powders. The microstructural analysis revealed the formation of the Fe_3_W_3_C phase in the reinforcement. The volume fraction of Fe_3_W_3_C particles increased with the Ni60WC25 amount (up to 35 vol%), and, simultaneously, an extensive dissolution of WC particles occurred, which decreased the wear resistance. Zhang et al. [16] fabricated a composite coating of WC-Hadfield steel and concluded that the WC particles partially dissolved and Fe_3_W_3_C and M_23_C_6_ phases precipitated in the composite zone. Later, Zhang et al. [19] produced a similar composite reinforcement with a medium carbon steel as base alloy; in this study, a 7 mm thick composite zone was formed, with about 32 vol% WC, and with a hardness 2.4 times greater than the steel. Regarding local reinforcement in low carbon steels, Sobula et al. [23] produced a TiC-steel composite coating using an *in situ* approach, increasing wear resistance, measured by weight loss, by four to six times compared to the cast alloy. In a more recent study, Olejnik et al. [22] investigated the addition of Fe to a Ti and C powder mixture to produce a TiC-low carbon steel composite and concluded that the addition of 30 wt.% Fe enabled control of the quality of the reinforcement resulting in the best hardness.

The main objective of this study is to improve the surface wear resistance of a low carbon cast steel, GP280GH ISO 4991 [24], with a metal matrix composite reinforced with WC (WC–MMC) to obtain a new material effective in competing with hard alloy steels along with its excellent weldability, being this topic slightly explored up to now, and representing one innovative solution. This work presents a comprehensive characterization of the phases formed in the composite zone, which is a key issue for obtaining high-quality reinforced components. For this purpose, mixtures of WC and Fe powders were used, the Fe powder acting as a flux to improve infiltration between the molten metal and the WC particles.

## 2. Materials and Methods

### 2.1. Production of the Reinforced Cast Specimens

The reinforced specimens were fabricated using the pressureless casting technique described in detail in one of our previous studies [25]. This procedure involved the following main steps:

1. Selection and weighing of Fe (99.0 wt.% purity) and WC (99.0 wt.% purity) powders from Alfa Aesar, ThermoFisher (Kandel, Germany) GmbH, fully characterized in the cited reference [25];
2. Mixing and homogenization of Fe and WC powders (in a volume fraction of 60:40) in a Turbula shaker–mixer (Willy A. Bachofen AG, Muttenz, Switzerland) for 7 h;
3. Cold pressing of the mixture; in this step, the mixture of powders was uniaxially cold-pressed at 230 MPa in a metallic mold to produce green compacts with a parallelepiped shape (31 mm × 12 mm × 7 mm);
4. Casting: at this step, the green compacts were inserted in specific locations of the mold before the pouring of the molten low carbon steel at 1620 °C; the chemical composition of the metal was analyzed by optical emission spectrometry (MAXx LMM05, Spectro, Germany) and was in correspondence with the ISO 4991 standard [24], presented in Table 1;
5. Normalization heat treatment; cylindrical specimens with 45 mm diameter were cut by electrical discharge machining to obtain 5-milimeter-thick samples that were heat-treated at 930 °C for 30 min as specified in ISO 4991 standard [24]; this is a common heat treatment for stress relief and structure refining of the cast components.

### 2.2. Microstructural Characterization

After the metallographic preparation of the samples and chemical etching with 2% Nital and Vilella’s reagent, the microstructure was characterized by scanning electron microscopy (SEM) with energy dispersive spectroscopy (EDS), using a FEI Quanta 400 FEG and a FEI Quanta 650 FEG (FEI Company, Hillsboro, OR, USA), both with an energy-dispersive detector. Electron backscatter diffraction (EBSD) analysis has been used to assist with phase identification. To complement the microstructural characterization, X-ray diffraction (XRD, Cu Kα radiation, Bruker D8 Discover, Billerica, MA, USA), with a scanning range (2θ) of 5° to 80° was performed. The volume percentage of carbides in the composite was measured using ImageJ (v.1.52, Wayne Rasband, National Institutes of Health, Bethesda, MD, USA), an open source image analysis software, and EDS maps at 500× magnification, since OM and BSE-SEM images did not provide adequate contrast to distinguish carbides from the matrix.

### 2.3. Mechanical Characterization

The mechanical response of the reinforced specimens was evaluated by hardness and ball-cratering abrasion tests. Seven Vicker’s hardness tests were performed on each specimen, according to the ISO 6507-1:2018 standard [26], applying a nominal force of 294.2 N in a universal hardness tester DuraVison 20 (EMCO-TEST Prüfmaschinen GmbH, Kuchl, Austria). Additionally, hardness profiles across the composite zones were made using a nominal force of 49.0 N.

The ball-cratering tests were carried out in a Plint TE66 micro-scale abrasion tester (Plint & Partners Ltd., Newbury, UK), according to the ISO 26424:2008 standard [27], as described in a previous study [28]. The specimens with a thickness of 5 mm were tested using a slurry of SiC abrasive particles on a rotating steel ball bearing and sliding distances of 7.9, 15.7, 23.6, and 31.4 m.

The wear craters were measured by optical microscopy (OM) using a Leica DM4000 M with a DMC 2900 camera (Leica Microsystems, Wetzlar, Germany) and image processing software (ImageJ v.1.52) and analyzed by SEM.

## 3. Results and Discussion

### 3.1. Microstructural Characterization of the Reinforced Cast Specimens

**Figure 1:**
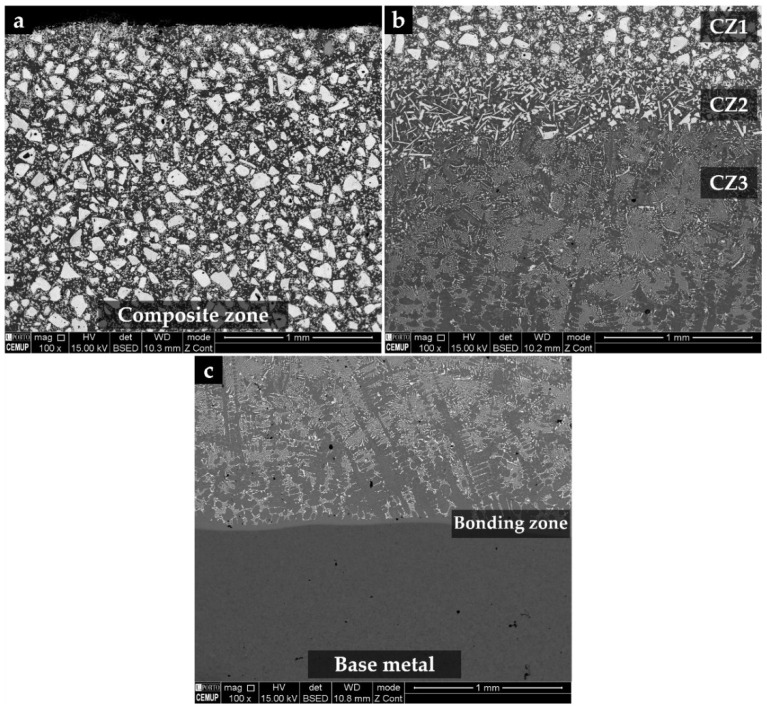
SEM–BSE images of the microstructure of the reinforced specimen, showing: CZ1—nearest to the surface (**a**), a transition region, with three distinct zones: CZ1, CZ2—intermediate, and CZ3—next to the base metal (**b**), and the interface with the steel (**c**).

**Figure 2:**
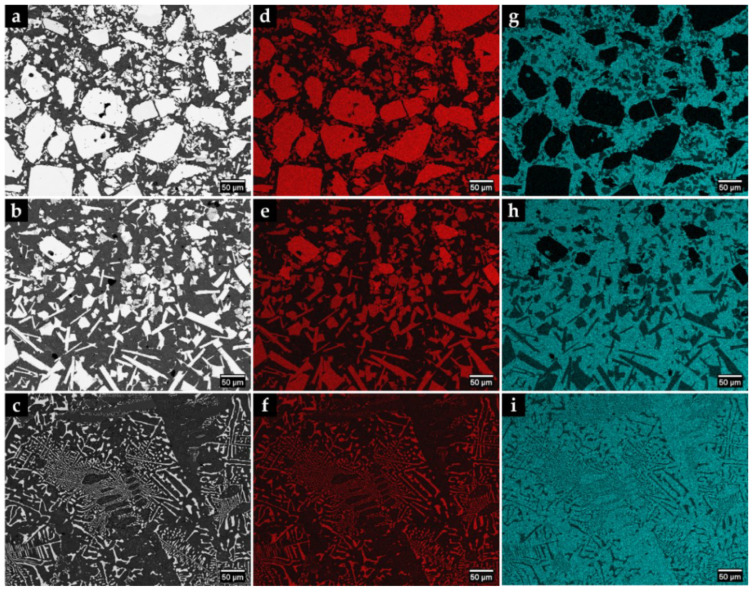
SEM–BSE images of the microstructure of the CZ1 (**a**) and CZ2 (**b**) and CZ3 (**c**). EDS elemental mapping (**d**–**i**) of W (red) and Fe (blue).

The microstructure of the composite and its interface with the base metal is shown in Figure 1. Three distinct regions are observed in the composite. The region next to the surface (CZ1), with a depth of 5.4 mm, exhibits a large number of original WC particles with a polygonal shape, as confirmed by EDS in Figure 2. Below the CZ1, a narrower region (CZ2) with a depth of 0.5 mm is characterized by plate shape particles rich in tungsten (W) and Fe (Figure 1b and Figure 2). The CZ3 zone, with a depth of 1.5 mm, shows a dendritic microstructure composed of W- and Fe-rich precipitates (see Figure 1b and Figure 2). The interface between the composite and the base metal is clearly distinguished in Figure 1c, showing good bonding without voids or discontinuities along with its thickness of 0.3 mm.

**Figure 3:**
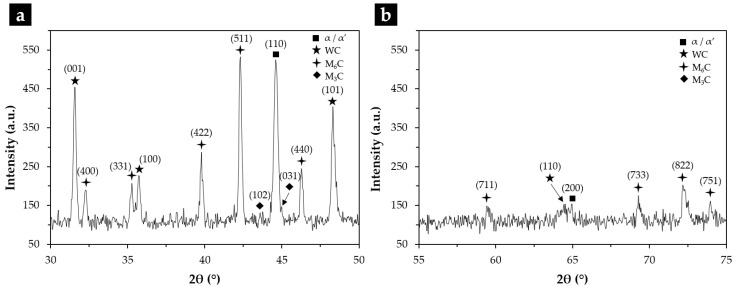
XRD patterns of the composite in the 2θ range of 30°–50° (**a**) and 50°–80° (**b**), identifying the type of carbides and other phases present.

XRD analysis permitted to identify the phases present in the microstructure, revealing several types of carbides (M_6_C and M_3_C) in addition to the original WC particles (Figure 3).

**Figure 4:**
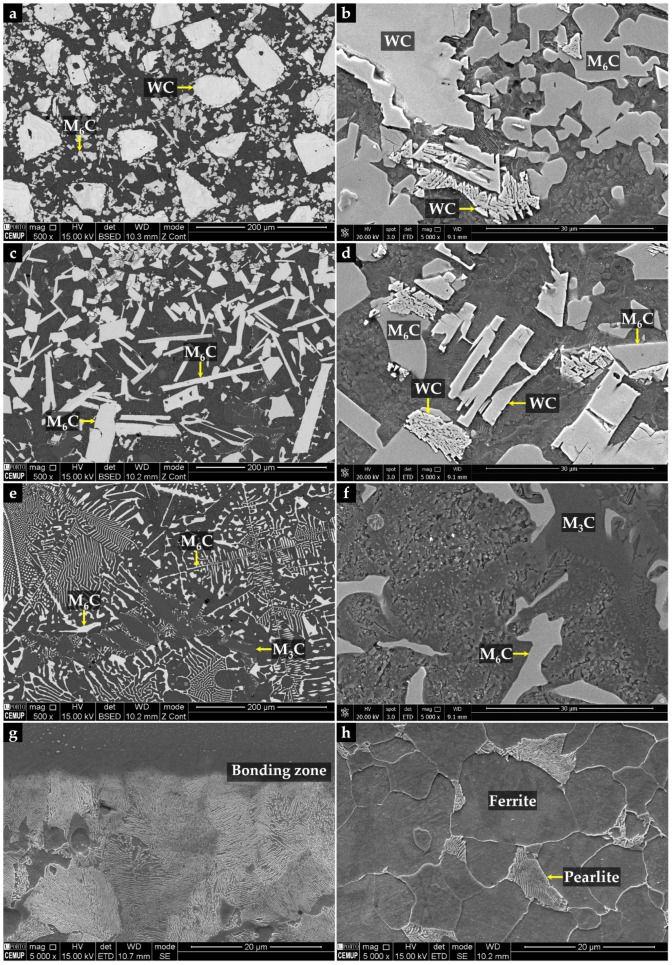
SEM images of the microstructure of the WC–MMC: CZ1 with a high content of original WC particles (**a**); (Fe,W)_6_C precipitation next to the WC particles (**b**); CZ2 evidencing a high number of (Fe,W)_6_C carbides with plate shape (**c**) and growth of (Fe,W)_6_C from the WC particles partially dissolved (**d**); CZ3 exhibiting essentially dendritic eutectic carbides of (Fe,W)_6_C and some massive carbides of (Fe,W)_3_C (**e**), and the same at higher magnification (**f**); bonding interface, showing the pearlite lamellar structure (**g**), and the base metal with a ferritic microstructure with a few pearlite colonies (**h**).

**Figure 5:**
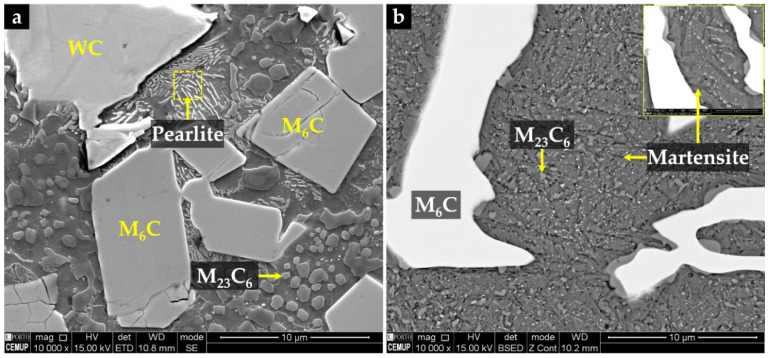
SEM–SE image of the CZ1, showing the α matrix with small colonies of lamellar pearlite and globular precipitation of (Fe,W)_23_C_6_ (**a**); and SEM–BSE image of the CZ3, evidencing the matrix of martensite, revealed with Vilella’s reagent, with a fine precipitation of (Fe,W)_23_C_6_ (**b**).

**Figure 6:**
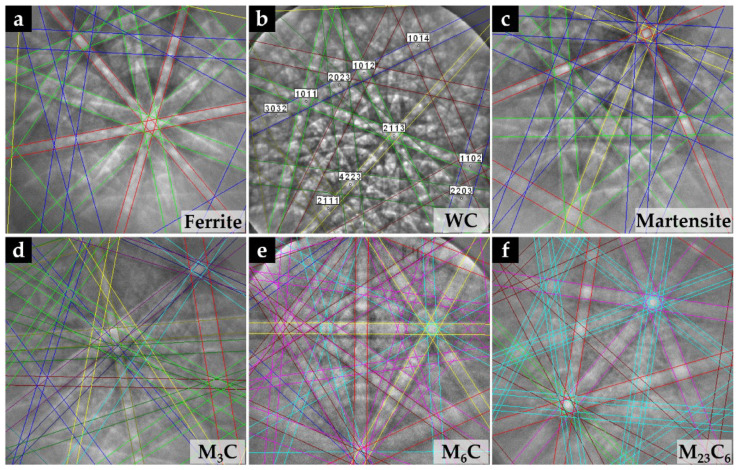
Indexed EBSD patterns corresponding to the phases present at the composite: ferrite (α) (**a**) and WC (**b**), both detected in the CZ1 and CZ2 composite zones; martensite (α’) found in the CZ3 region (**c**); and (Fe,W)_3_C (**d**), (Fe,W)_6_C (**e**) and (Fe,W)_23_C_6_ (**f**) detected in the matrix of the composite.

The properties of the resulting metal matrix composite reinforced with WC (WC–MMC) are determined by the morphology and distribution of the phases in the microstructure and, therefore, a detailed SEM analysis was performed (see Figure 4 and Figure 5). The SEM analysis included using the EBSD characterization technique to corroborate the phase identification (Figure 6). The CZ1 region exhibits a high content of original WC particles (in white), homogeneously dispersed in the matrix, as indicated in Figure 4a,b. It is clear that some of those particles have partially dissolved, acting as nucleation sites for the (Fe,W)_6_C precipitation at the interface of WC particles and the matrix. The (Fe,W)_6_C particles were identified by means of EBSD, as shown in Figure 6e. This is in line with the findings from a study on WC–MMC produced by laser melt injection, where (Fe,W)_6_C is formed by a peritectic reaction involving the liquid substrate (steel) and the injection WC particles [29]. Other studies [14,16,25] also reported this occurrence. The percentage of (Fe,W)_6_C along with WC corresponds to 59% of the CZ1 composite area.

In the CZ2 zone, there are only a few WC particles (Figure 4c,d). The greater dissolution of the original WC particles could have arisen from the higher temperature in this region that is further away from the cold mold wall. Consequently, a high number of plate-shaped (Fe,W)_6_C carbides (Figure 4d) have formed as described for the CZ1 zone. Comparatively, the total percentage of carbides is lower and corresponds to 36% of the CZ2 area.

The CZ3 region shows a much more complex microstructure. The SEM images indicate the absence of WC particles, meaning that the present phases were formed from a liquid enriched in W and carbon (C) due to the dissolution of the original WC particles. The microstructure exhibits essentially dendritic eutectic carbides of (Fe,W)_6_C and some massive carbides of (Fe,W)_3_C. These phases were confirmed by EBSD, as shown in Figure 6d,e. The dendritic morphology of the (Fe,W)_6_C phase was also showed by Sui et al. [14]. Regarding their quantification, since the massive carbides exhibit a contrast similar to the matrix, it was only possible to measure the dendritic eutectic carbides, corresponding to 19% of the composite area.

Concerning the matrix of CZ1 and CZ2, small colonies of lamellar pearlite are present, as shown in Figure 5. The EBSD patterns (Figure 6a) also indicate the presence of ferrite in these regions.

According to the CZ3 region, the matrix is essentially martensite (Figure 5b), as confirmed by EBSD analysis (Figure 6c). Such matrix variation along the reinforcement region can be explained by the progressive enrichment in C and W across its depth. This enrichment, which results from the dissolution of the original WC particles, suppresses ferrite formation and triggers the martensitic transformation [30,31]. In addition, small globular particles have precipitated in the matrix, which were identified as (Fe,W)_23_C_6_ by the EBSD technique (Figure 6f). The size and shape of these particles suggest this precipitation occurred during the post-casting heat treatment and which is expected to increase the hardness of the composite.

The microstructure of the interface between CZ3 and the base metal is essentially composed of pearlite, promoted by the C and W enrichment of the base metal (Figure 4g). This region with intermediate characteristics between the CZ3 and the base metal contributes to a smooth change of the properties, having a beneficial effect on the integrity of the reinforcement. Finally, the base metal shows the typical microstructure of a low carbon cast steel composed of ferrite with a few pearlite colonies (Figure 4h).

### 3.2. Mechanical Characterization of the Composite Reinforcement

#### 3.2.1. Hardness Results

**Figure 7:**
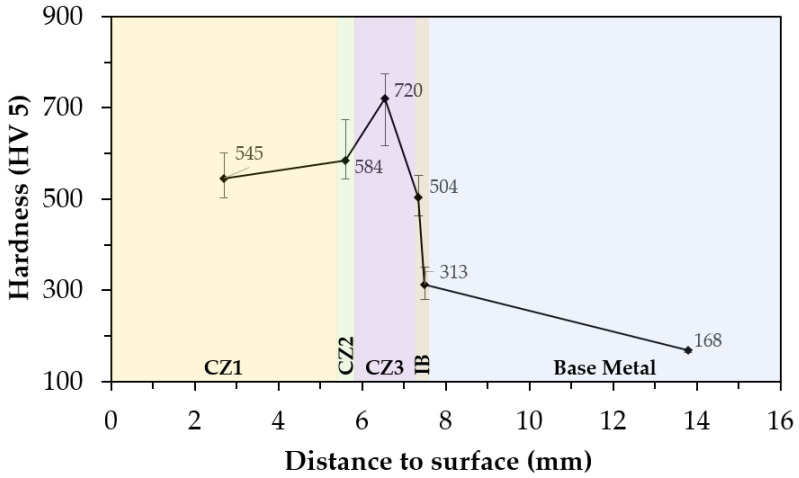
Hardness profile from the composite surface (CZ1) to the base metal.

The hardness of the WC–MMC next to the surface (CZ1) is three times higher than the hardness of the base metal, 504 HV 30 and 161 HV 30, respectively. This result is in line with the study by Zhang et al. [19] on medium carbon steel that reported a hardness of the composite zone 2.4 times harder than that of the base steel. A large variation in hardness is also found along with the depth of the composite due to the microstructural variation. According to the hardness profile shown in Figure 7, the highest values observed in the CZ3 zone (720 HV 5) are associated with the eutectic precipitation of (Fe,W)_6_C and fine precipitation of (Fe,W)_23_C_6_ in the martensite matrix. The hardness decrease from this region to the interface zone was attributed to the significant reduction of carbides precipitation. At the interface bonding, the hardness is significantly higher when compared to the base metal, explained by the pearlitic structure.

#### 3.2.2. Abrasion Wear Behavior

**Figure 8:**
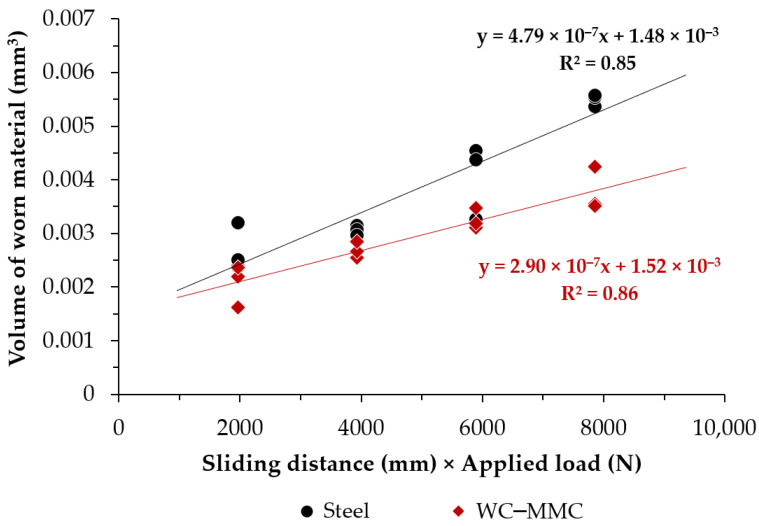
Volume of worn material as a function of the sliding distance under a constant load of 0.25 N for the WC–MMC and base metal.

The results of the micro-abrasion wear test are shown in Figure 8 and refer only to the CZ1 zone of the WC–MMC and the base metal since it was not possible to perform tests on CZ2 and CZ3 due to their small dimensions. The volume of worn material V (mm^3^), calculated using Equation (1), increases by increasing the sliding distance S (mm). However, the increase is less pronounced for the WC–MMC than for the base metal.

The Archard equation (Equation (2)) [32], and Czichos approach [33], were applied to the analysis of the results, leading to the wear rate coefficient K (mm^3^N^−1^mm^−1^) obtained from the slope of the straight line best adjusted to the experimental data. The WC–MMC showed a wear rate 39% lower than the base metal.

(1) $$V=\pi \times \frac{b^{4}}{64\times R}\;b:\;crater\;mean\;diameter\;\left(mm\right);\;R:\;steel\;ball\;radius\;\left(mm\right)$$

(2) $$K=V\times \frac{1}{S\times N}\;\;\;\;\;\;\;\;\;\;\;\;\;\;\;\;\;\;N:\;applied\;load$$

**Figure 9:**
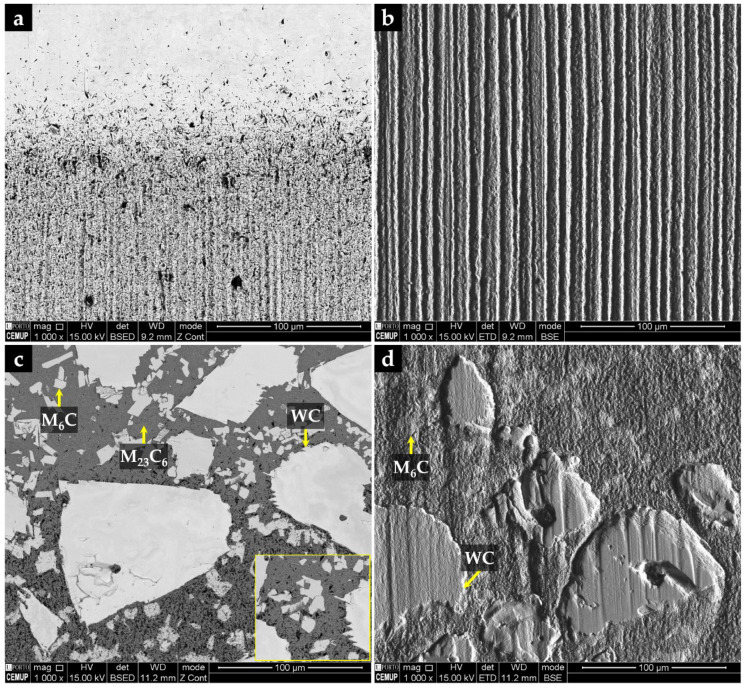
SEM images of the wear craters after a sliding distance of 31.4 m: (**a**,**b**) base metal, (**c**,**d**) the WC–MMC; (**a**,**c**) are regions close to the edge of the crater, and (**b**,**d**) are regions of the surface of the crater.

SEM analyses of the wear craters were performed to help the understanding of the wear mechanisms involved. From the images in Figure 9, it is clear that the WC particles resist the abrasion wear effectively, seeming that they have a protective effect against the deformation and wear of the matrix. Similar behavior was observed in WC–composite reinforcements on high carbon chromium steel [14], and Hadfield steel [16]. In addition, the images do not reveal any pulled-out traces, indicating a good quality bonding between WC particles and the matrix. The effect of smaller carbides on wear behavior is hard to evaluate; however, (Fe,W)_6_C particles seem to be in relief upon the surrounding matrix. The protective effect of the (Fe,W)_6_C phase was also reported by Sui et al. [14]. On the other hand, a huge amount of grooves evenly spaced are seen on the worn surface of the base metal (Figure 9a,b), indicating that plastic deformation is the predominant wear mechanism owing to the soft ferritic–pearlitic microstructure.

## 4. Conclusions

A low carbon cast steel was successfully reinforced with WC–MMCs, using a Fe–WC preform prepared from a mixture of WC and Fe powders in a volume ratio of 40:60.

A particular characteristic of this WC–MMC reinforcement is the microstructural variation throughout its depth, explained by the dissolution of the WC particles in the liquid metal, leading to the precipitation of (Fe,W)_6_C carbides and, closer to the base metal, dendritic carbides of (Fe,W)_6_C and fine (Fe,W)_23_C_6_ particles. The formation of graded material, with microstructural and hardness gradients across the reinforcement and the interface with the base metal, ensures good structural integrity of the resulting reinforcement.

This approach provided a wear rate decrease of 39%, and it may be well suited for low carbon cast steel applications requiring wear surfaces for working under harsh conditions.

The results of this work evidenced high potential for industrialization in foundry companies, although the feasibility of applying other powders’ ratio and their effect on the mechanical properties can be studied in the future.

## Figures and Tables

**Table 1:** Nominal chemical composition (wt.%) of the low carbon cast steel [20].

C	Si	Mn	Cr	Ni	Cu	Fe
0.22	0.43	0.91	0.10	0.09	0.03	Balance

## Data Availability

Not applicable.
